# Combination Therapy of Intense Pulsed Light and Fractional Carbon Dioxide Laser Versus Botulinum Toxin Type A in Post-thyroidectomy Scar Prevention: A Prospective, Randomized Controlled Trial

**DOI:** 10.1007/s00266-026-05717-2

**Published:** 2026-03-25

**Authors:** Rong Xin Ren, Wen Jiang Qian, Hong Yi Zhao, Tian Ming Ma, Dong Dong Wang, Jian Kun Li, Gang Miao

**Affiliations:** 1 Department of Plastic Surgery, Beijing Hospital, National Center of Gerontology, Institute of Geriatric Medicine, Chinese Academy of Medical Sciences, Beijing, China; 2 Department of General Surgery, Beijing Hospital, National Center of Gerontology, Institute of Geriatric Medicine, Chinese Academy of Medical Sciences, Beijing, China

**Keywords:** Intense pulsed light, Fractional carbon dioxide laser, Botulinum toxin type A, Randomized controlled trial, Scar prevention, Thyroidectomy

## Abstract

**Objective:**

This study compared the efficacy and safety of botulinum toxin type A (BTX-A) injection and CO_2_ ablative fractional laser combined with intense pulsed light (CO_2_AFL-IPL) therapy in preventing hypertrophic scarring after thyroidectomy.

**Approach:**

In this single-center, prospective, randomized controlled trial, 105 patients undergoing open thyroidectomy were assigned to three groups: silicone dressing (control), CO_2_AFL-IPL therapy, or BTX-A injection. Scar outcomes were assessed over 12 months using the Patient and Observer Scar Assessment Scale (POSAS), modified Vancouver Scar Scale (mVSS), 3D imaging, colorimetry, and adverse event reports.

**Results:**

Both BTX-A and CO_2_AFL-IPL groups showed significantly improved POSAS and mVSS scores compared to the control group at 6 and 12 months (*p* < 0.001), with no significant difference between the two treatment arms. BTX-A treatment had fewer adverse events and lower pain scores. Colorimetry and 3D imaging revealed no significant differences between groups.

**Conclusion:**

Both BTX-A and CO_2_AFL-IPL effectively prevented post-thyroidectomy scarring, each outperforming silicone dressings. While their efficacy was comparable, BTX-A offered greater convenience, safety, and patient comfort, making it a more practical option for routine clinical use, with CO_2_AFL-IPL serving as an effective alternative where laser therapy is available.

**Level of Evidence I:**

This journal requires that authors assign a level of evidence to each article. For a full description of these Evidence-Based Medicine ratings, please refer to the Table of Contents or the online Instructions to Authors www.springer.com/00266.

## Introduction

Thyroid carcinoma is the most frequently diagnosed endocrine system malignancy worldwide. In 2022, approximately 821,000 new cases were reported globally, with incidence rates demonstrating an upward trend and projected to increase by 44.1% between 2019 and 2030. The disease predominantly affects women, with incidence rates three to four times higher than those in men, accounting for approximately 74.86% of all cases [1]. Thyroidectomy procedures result in surgical wounds on the anterior neck, a prominently visible anatomical location. Postoperative scarring in this area can markedly influence the overall quality of life of affected individuals, particularly among female patients who often express greater concern regarding cervical aesthetics [2].

Wound healing comprises three overlapping yet distinct biological phases: inflammation, proliferation, and remodeling. During thyroidectomy, scar formation, abnormal vascularization, imbalanced collagen deposition, and persistent mechanical tension are the primary mechanisms driving pathological scarring [3]. Early preventive interventions targeting these critical phases may enhance final scar quality through modulation of inflammatory responses and collagen remodeling processes. Current scar prevention strategies include topical silicone preparations, botulinum toxin A (BTX-A) injections, and laser therapies [4, 5]. Although surgical wound closure techniques in thyroidectomy patients have been systematically assessed, with evidence showing notable differences in scar appearance and healing time among closure methods [6], current research increasingly focuses on postoperative strategies for scar prevention and remodeling. BTX-A is thought to improve scar outcomes primarily by inhibiting acetylcholine release at the neuromuscular junction, thereby reducing dynamic wound-edge tension during the early remodeling phase, with effects lasting approximately 3–6 months [7]. In addition to conventional intramuscular injection, microdosed BTX techniques (“Microbotox”), involving superficial rather than intramuscular delivery, have also been described in hypertrophic and keloid scar management [8, 9]. Laser therapies, particularly CO_2_ ablative fractional laser combined with narrowband intense pulsed light therapy (CO_2_AFL-IPL), target different aspects of scar pathophysiology through distinct mechanisms. The CO_2_ fractional laser creates microscopic thermal treatment zones to promote collagen remodeling and tissue regeneration [10]. Meanwhile, narrowband intense pulsed light (500–600 nm) concentrates light energy within a narrow spectrum. It specifically targets the absorption wavelengths of hemoglobin at 542 and 577 nm to coagulate the microvasculature while minimizing nonspecific thermal damage to nearby tissue [11]. Despite the demonstrated efficacy of these treatment modalities in early scar prevention, most prior studies have evaluated BTX-A and laser-based approaches separately or have compared single-laser protocols; direct head-to-head evidence in the early post-thyroidectomy prevention window—particularly contrasting BTX-A with a protocolized CO_2_AFL-IPL combination—remains limited.

Therefore, in this randomized, prospective trial, we aimed to compare the safety and effectiveness of two commonly adopted early intervention strategies, namely BTX-A injection and a protocolized CO_2_AFL–IPL regimen, for preventing post-thyroidectomy scar formation.

## Methods

### Trial Design

This single-center, prospective, randomized, parallel-group clinical trial was conducted at Beijing Hospital. A total of 105 patients requiring open thyroidectomy for thyroid malignancy were recruited from the Departments of Plastic Surgery and General Surgery between September 2023 and April 2024. The sample size was calculated using PASS 21.0 based on the primary endpoint (6-month POSAS score). Previous study reported a mean post-treatment POSAS score of approximately 23 points in the control group [12]. Based on this, we assumed that the investigational treatments would reduce the score to at least 15 points, with a standard deviation of 11 points. With a two-sided α of 0.05 and 80% power, 29 participants were required per group. Considering potential loss to follow-up, the target sample size was increased to 35 per group, yielding a total of 105 participants. Eligible participants met the following criteria: 18–65 years of age; had undergone open thyroidectomy within the past week; were fully informed of the treatment protocol and potential risks; agreed to participate in follow-up, photography, and noninvasive skin assessments; voluntarily participated in this clinical research; and provided written informed consent. Participants were excluded if they met any of the following criteria: swallowing or breathing difficulties, peripheral motor neuropathy or unhealed wounds in the cervical surgical area; local skin infections or ulcerations; women who were pregnant, breastfeeding, or planning to become pregnant; had neuromuscular dysfunction or severe organic or psychiatric disease; or concurrent participation in other clinical trials.

### Ethical Approval

This study was approved by the Ethics Committee of Beijing Hospital (Approval No. 2023BJYYEC-259-02) and registered with the Chinese Clinical Trial Registry (ChiCTR2300075601). All procedures adhered to the principles of the Declaration of Helsinki. Each participant provided written informed consent after fully understanding the research objectives, methods, potential risks, and benefits. All included participants agreed to comply with the treatment and follow-up protocols and granted permission for the use of research materials, such as photographs, for scientific publication.

### Random Allocation and Group Assignment

A stratified randomization method was employed, with scar risk assessment (high, moderate, or low) used as the stratification factor. The criteria for risk classification followed the standards for high-risk scar factors outlined in the Chinese Clinical Expert Consensus on Scar Prevention and Treatment [13]. After confirming eligibility, the 105 qualified patients randomly assigned in equal proportions (1:1:1) to three study arms: Groups A (control), B (CO_2_AFL-IPL), and C (BTX-A), with 35 patients in each group. The randomization sequence was computer-generated using stratified permuted blocks by an independent statistician who was not involved in patient enrollment or treatment. Patient allocation strictly followed this pre-generated sequence.

### Blinding and Bias Control

Allocation concealment was ensured using sealed, opaque envelopes prepared and managed by an independent coordinator who was not involved in treatment or evaluation. Because the interventions were visually distinguishable, blinding of patients and treatment providers was not feasible. However, to minimize detection bias, outcome assessors were blinded to group allocation. POSAS and mVSS ratings were performed during face-to-face outpatient visits by two clinicians not involved in treatment and not informed of group allocation. Standardized, anonymized photographs were taken at each visit to document scar appearance and were used only as supplementary material to support inter-rater consistency, not as the primary basis for scoring. In addition, objective assessments using 3D imaging and colorimetry followed predefined protocols to further reduce subjective bias.

### Treatment Protocol

Patients who met the eligibility criteria and underwent open thyroidectomy at the Department of General Surgery, Beijing Hospital, between September 2023 and April 2024, were recruited. The operations were performed in accordance with standardized surgical guidelines by a surgical team led by Chief Surgeon Gang Miao, with Drs. Dongdong Wang, Jiankun Li, and Tianming Ma participating. A cervical collar incision (5–7 cm in length) was made along the skin creases. The skin, subcutaneous tissue, and platysma were dissected layer by layer, and the strap muscles were divided to expose the thyroid capsules. After completion of the thyroidectomy and hemostasis, a closed-suction drainage system was placed. The layer-by-layer moderate tension reduction suture technique was performed using 3-0 absorbable sutures and 3-0 prolene sutures to minimize postoperative scarring [14, 15].

Following early scar-management recommendations, all patients began intervention therapy 2 weeks postoperatively.

For Group A, patients received standard care with silicone sheeting (Mepiform®, Mölnlycke Health Care, Sweden, Registration No. 201626422805) applied topically for 12 h daily. The sheets were trimmed to slightly exceed the wound margins and were administered continuously for up to 6 months.

For Group B, patients received a combined CO_2_AFL–IPL treatment according to a predefined protocol. Narrowband IPL treatment was performed by applying a 2–3 mm layer of ultrasound gel over the wound area, followed by irradiation using a commercial IPL system (Alma Laser, Caesarea, Israel) at a wavelength of 500–600 nm, 3 cm^2^ spot size, fluence of 10–12 J/cm^2^, pulse width of 12 ms, and 2 passes. Subsequently, CO_2_AFL therapy was performed using the Deep FX mode of a fractional CO_2_ laser system (Lumenis, Santa Clara, CA, USA), delivering 10–15 mJ/cm^2^ in a single pass at 10% density. The treatment endpoint was characterized by visible tissue ablation with mild erythema and edema. After each session, patients applied topical antibacterial ointment and moisturizer twice daily for 7 days, maintained wound hygiene, avoid strenuous activities and saunas for 1 week, and use sun-protective measures. Three sessions were completed at 4-week intervals.

For Group C, BTX-A (100 U/vial; Lanzhou Biochemical Company, Lanzhou; National Medicine Approval No. S10970037) was diluted in normal saline to prepare a 4 U/0.1 mL solution. Injections were administered on either side of the wound at 1–1.5 cm intervals, with 4 U administered at each injection site using a 1 mL syringe (29G needle). The total dose per treatment session was 30 U.

### Subjective and Objective Assessments

Assessment time points were set at 1, 3, 6, and 12 months postoperatively. The primary outcome was the Patient and Observer Scar Assessment Scale (POSAS) score assessed at the 6-month follow-up. Secondary outcomes included evaluations using the Observer Scar Assessment Scale (OSAS), Patient Scar Assessment Scale (PSAS), POSAS, and the modified Vancouver Scar Scale (mVSS) at other time points. All evaluations were independently performed by two experienced plastic surgeons who were not involved in treatment. Both assessors received standardized training before the study and were blinded to treatment allocation. All scar assessments were conducted during face-to-face outpatient visits through direct visual inspection and palpation, accompanied by structured questioning about subjective symptoms. Standardized, anonymized photographs were taken at each visit to document scar appearance and to support inter-rater consistency checks.

POSAS includes the OSAS and PSAS. The OSAS evaluates physical features such as vascularity, pigmentation, relief, and pliability, while the PSAS captures subjective symptoms, including pain, itching, and stiffness. Each item is rated from 1 (normal) to 10 (worst imaginable scar). The mVSS assesses pigmentation, vascularity and thickness (each scored 0–3), pliability (0–5), pain and itching (each scored 0–2), with higher scores indicating worse outcomes. The Numeric Rating Scale (NRS) was employed to evaluate procedural pain in participants from Groups B and C.

Objective assessments included three-dimensional imaging using the Vectra H2 3D Imaging System, with analysis performed using Vectra software (Canfield Scientific, Inc., Parsippany, NJ, USA). Scar pigmentation and vascularity were further assessed using narrowband reflectance spectrophotometry (DSM-IV; Cortex, Hadsund, Denmark).

### Data Analysis

Data were analyzed using SPSS software (version 26.0; SPSS Inc., San Diego, CA, USA). Categorical variables are expressed as absolute frequencies and relative percentages and were compared between groups using the Pearson chi-square or Fisher’s exact test when appropriate. Continuous variables meeting normality assumptions are presented as mean ± standard deviation (SD) and were compared using the F test. Non-normally distributed data are summarized as medians and interquartile ranges (M [p25, p75]), with intergroup differences analyzed via the Kruskal–Wallis H test. All statistical analyses were two-sided, with significance defined as *p* < 0.05. The Bonferroni method was employed to correct α for multiple comparisons.

To address missing data, multiple imputation was performed using the chained equations method in SPSS 26.0. Five imputed datasets were generated, with all analysis variables included in the imputation model. After imputation, estimates were combined using Rubin’s rules. Sensitivity analyses were performed to assess the robustness of the findings with respect to missing data handling.

## Results

### Patient Demographics

**Fig. 1:**
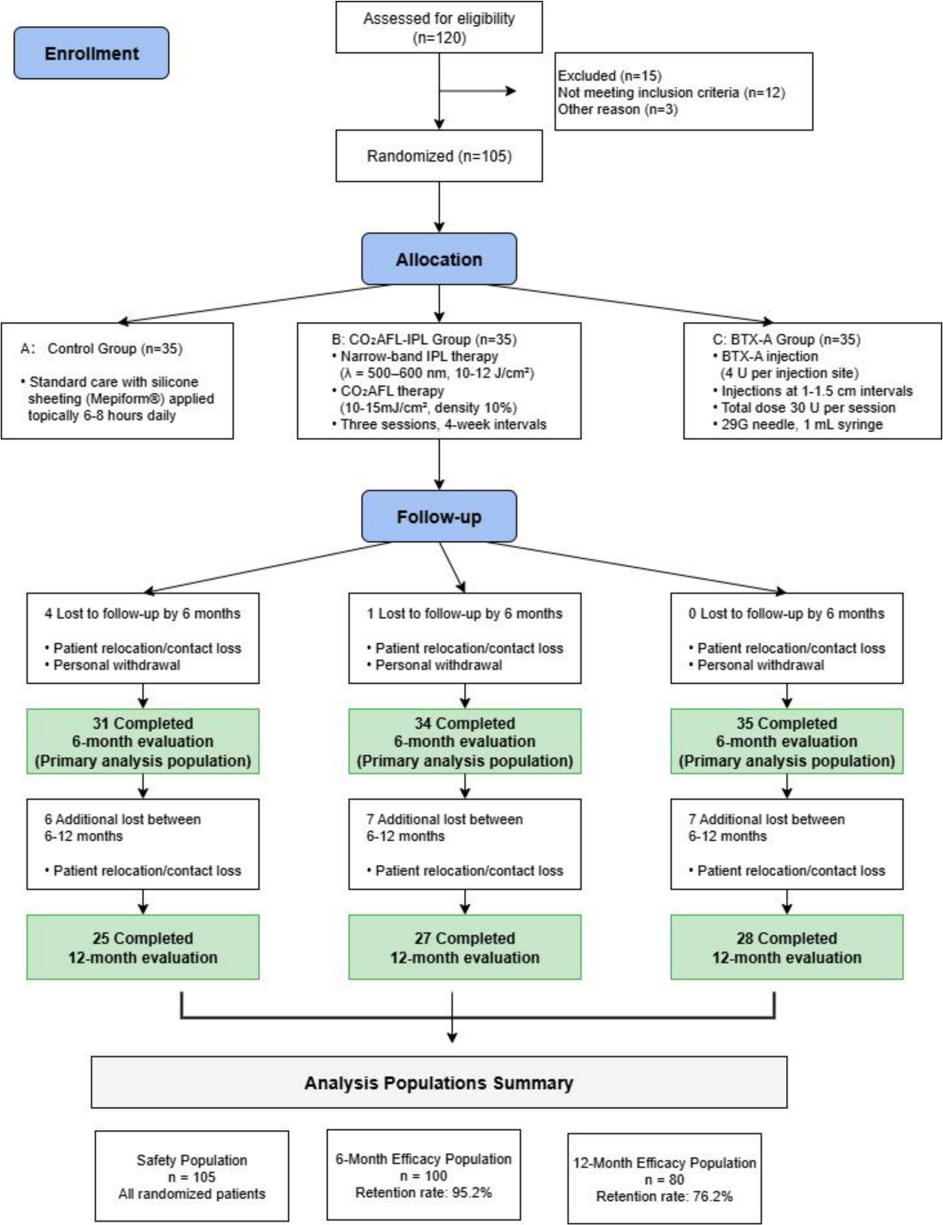
The consort flowchart of the clinical trial

Among the 120 patients initially assessed for eligibility, 105 met the inclusion criteria and were enrolled in the study. Of the 15 excluded patients, 12 did not meet the inclusion criteria, and the remaining three were excluded for other reasons. In a 1:1:1 randomization scheme, 105 patients were assigned to three arms: Groups A (control), B (CO_2_AFL-IPL), and C (BTX-A), with 35 patients in each. Among them, 100 completed scar evaluations at the 6-month follow-up, and 80 completed the 12-month follow-up (Fig. 1). Participant ages ranged from 18 to 65 years, with mean ages of 40.29 ± 6.83 years in Group A, 38.09 ± 8.00 years in Group B, and 37.37 ± 6.66 years in Group C. Overall, 98 participants were women (93.3%) and 7 were men (6.7%). Based on scar-formation risk stratification, 58 patients (55.2%) were classified as low risk and 47 (44.8%) as high risk. Baseline characteristics did not significantly vary across the groups (*p* > 0.05; Table 1).

**Table 1:** Study demographics

Variables	Control group	CO_2_AFL-IPL group	BTX-A group	P value
Age	40.29 ± 6.83	38.09 ± 8.00	37.37 ± 6.66	0.215
Sex				0.157
Male	4 (11.43)	0 (0.00)	3 (8.57)	
Female	31 (88.57)	35 (100.00)	32 (91.43)	
Risk				0.151
Low	24 (68.57)	17 (48.57)	17 (48.57)	
High	11 (31.43)	18 (51.43)	18 (51.43)	

### POSAS and mVSS Score Scar

**Fig. 2:**
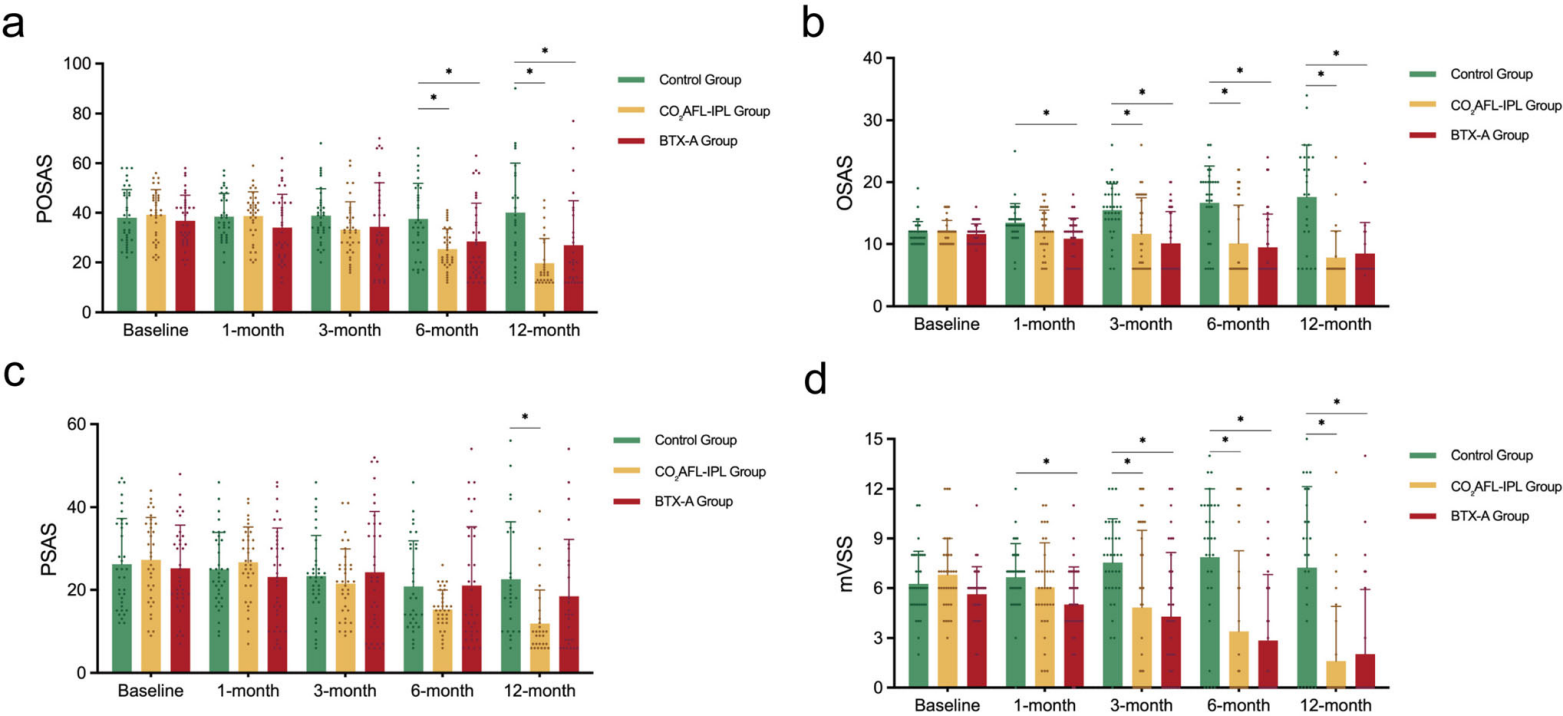
Comparison of scar assessment scores among the three treatment groups at different follow-up time points. **a** POSAS **b** OSAS **c** PSAS **d** mVSS

Scar assessment was conducted using the POSAS, OSAS, PSAS, and mVSS scales at 1-, 3-, 6-, and 12-month follow-ups (Fig. 2, Table 2). The primary endpoint was the 6-month POSAS total score, and the secondary endpoints included OSAS, PSAS, and mVSS scores at the same follow-up points.

**Table 2:** Scar assessment results at different follow-up time points

Scar assessment	Baseline			*P*	1-month			*P*	3-month			*P*
	Control group	CO_2_AFL-IPL group	BTX-A group		Control group	CO_2_AFL-IPL group	BTX-A group		Control group	CO_2_AFL-IPL group	BTX-A group	
POSAS	38.03 ± 11.34	39.17 ± 10.21	36.83 ± 10.26	0.654	38.43 ± 9.33	38.74 ± 9.73	34.03 ± 13.45	0.139	38.89 ± 10.84	33.20 ± 11.22	34.40 ± 17.71	0.189
OSAS	12.00	12.00	12.00	0.882	13.00	12.00	12.00	**0.005**	16.00	10.00	6.00	**< 0.001**
	(11.00,12.00)	(10.00,12.00)	(10.00,12.00)		(12.00,14.00)	(10.00,14.50)	(8.00,13.00)*		(14.00,18.00)	(6.00,18.00)*	(6.00,15.50)*	
PSAS	25.00	28.00	23.00	0.681	24.00	27.00	23.00	0.298	23.00	22.00	22.00	0.800
	(16.50,36.00)	(19.00,36.00)	(18.50,32.50)		(18.50,32.00)	(22.00,32.50)	(12.50,31.50)		(18.50,26.50)	(15.00,26.00)	(11.50,36.00)	
mVSS	6.00	7.00	6.00	0.060	7.00	6.00	5.00	**0.004**	8.00	5.00	4.00	**0.003**
	(5.00,8.00)	(5.50,8.00)	(5.00,6.00)		(6.00,7.50)	(5.00,7.00)	(4.00,6.50)*		(6.00,9.00)	(0.00,10.00)*	(0.50,7.00)*	

Scar assessment	Baseline			*P*	6-month			*P*	12-month			*P*
	Control group	CO_2_AFL-IPL group	BTX-A group		Control group	CO_2_AFL-IPL group	BTX-A group		Control group	CO_2_AFL-IPL group	BTX-A group	
POSAS	38.03 ± 11.34	39.17 ± 10.21	36.83 ± 10.26	0.654	37.52 ± 14.40	25.40 ± 8.11*	28.44±15.40*	**0.001**	40.16 ± 19.91	19.71 ± 9.93*	26.96 ± 17.94*	**< 0.001**
OSAS	12.00	12.00	12.00	0.882	18.00	6.00	6.00	**< 0.001**	18.00	6.00	6.00	**< 0.001**
	(11.00,12.00)	(10.00,12.00)	(10.00,12.00)		(12.00,20.00)	(6.00,18.00)*	(6.00,13.25)*		(12.00,24.00)	(6.00,6.00)*	(6.00,8.00)*	
PSAS	25.00	28.00	23.00	0.681	19.00	15.00	14.00	0.373	18.00	9.50	14.00	**0.003**
	(16.50,36.00)	(19.00,36.00)	(18.50,32.50)		(13.00,31.00)	(12.00,19.75)	(9.00,26.00)		(10.00,34.00)	(7.00,12.75)*	(7.00,27.00)	
mVSS	6.00	7.00	6.00	0.060	9.00	0.00	0.00	**< 0.001**	8.00	0.00	0.00	**< 0.001**
	(5.00,8.00)	(5.50,8.00)	(5.00,6.00)		(6.00,11.00)	(0.00,6.50)*	(0.00,6.00)*		(4.00,11.00)	(0.00,0.50)*	(0.00,1.50)*	

The bold values indicate statistically significant differences between groups at the corresponding follow-up time points

Data are presented as mean ± standard deviation or median (interquartile range). *indicates statistically significant difference compared to baseline (P < 0.05). *POSAS* Patient and Observer Scar Assessment Scale, *OSAS* Observer Scar Assessment Scale, *PSAS* Patient Scar Assessment Scale, *VSS* Vancouver Scar Scale, *BTX-A* Botulinum toxin A, and *CO*_*2*_*AFL-IPL* Carbon dioxide fractional laser combined with intense pulsed light.

**Fig. 3:**
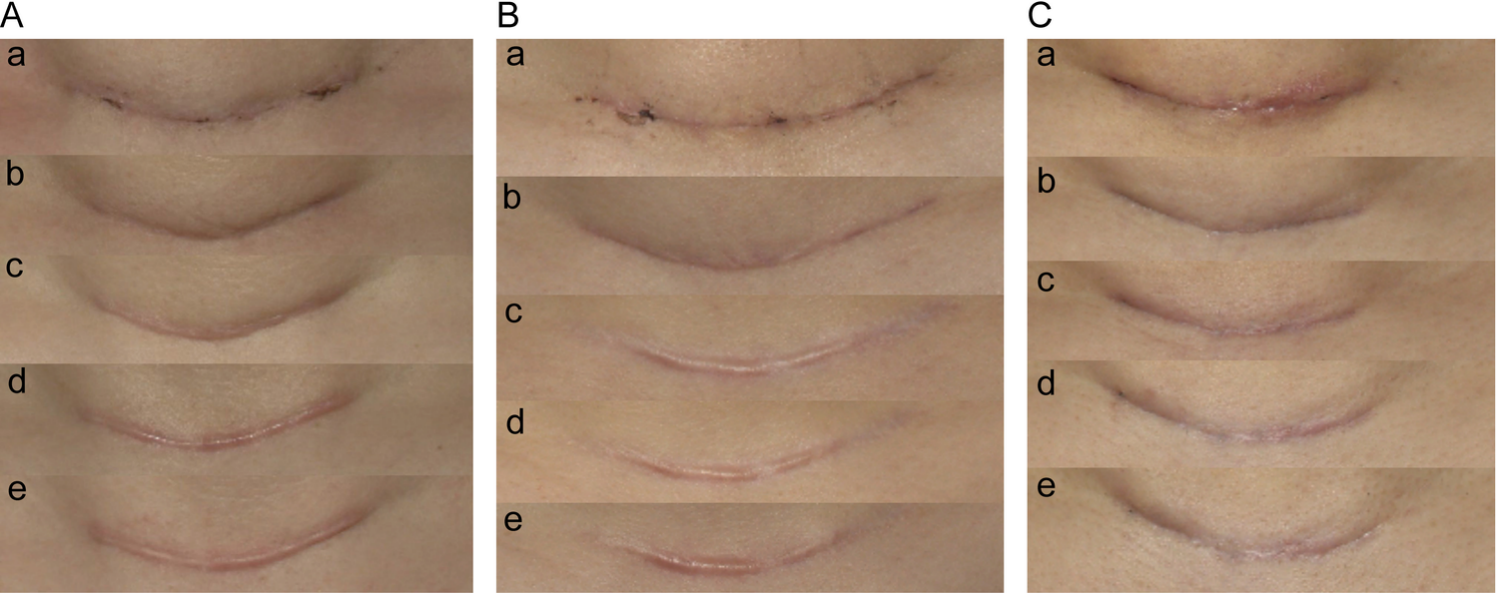
Representative scar healing progression showing worst-case scenarios across treatment groups during 12-month follow-up period. **A** Group A (silicone dressing), **B** Group B (CO_2_AFL-IPL), (**C**) Group C (BTX-A). Time points: **a** baseline, **b** 1 month, **c** 3 months, **d** 6 months, **e** 12 months postoperatively. Even in the worst-evaluated cases, both treatment groups demonstrated superior scar quality compared to the control group. All photographs were captured under standardized conditions with consistent lighting, fixed camera distance and angle, and identical exposure settings using a DSLR camera

**Fig. 4:**
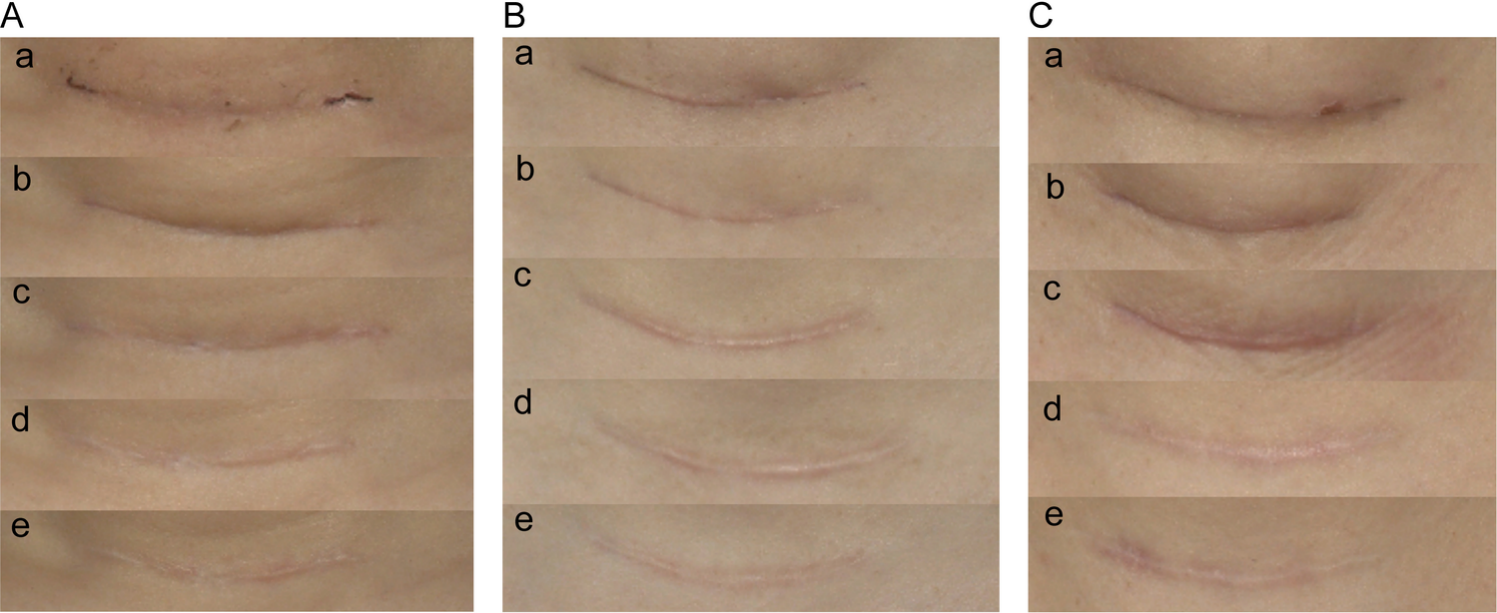
Representative scar healing progression showing moderate-case scenarios across treatment groups during 12-month follow-up period. **A** Group A (silicone dressing), **B** Group B (CO_2_AFL-IPL), **C** Group C (BTX-A). Time points: **a** baseline, **b** 1 month, **c** 3 months, **d** 6 months, **e** 12 months postoperatively. Photographic conditions were identical to those described for Fig. 3

**Fig. 5:**
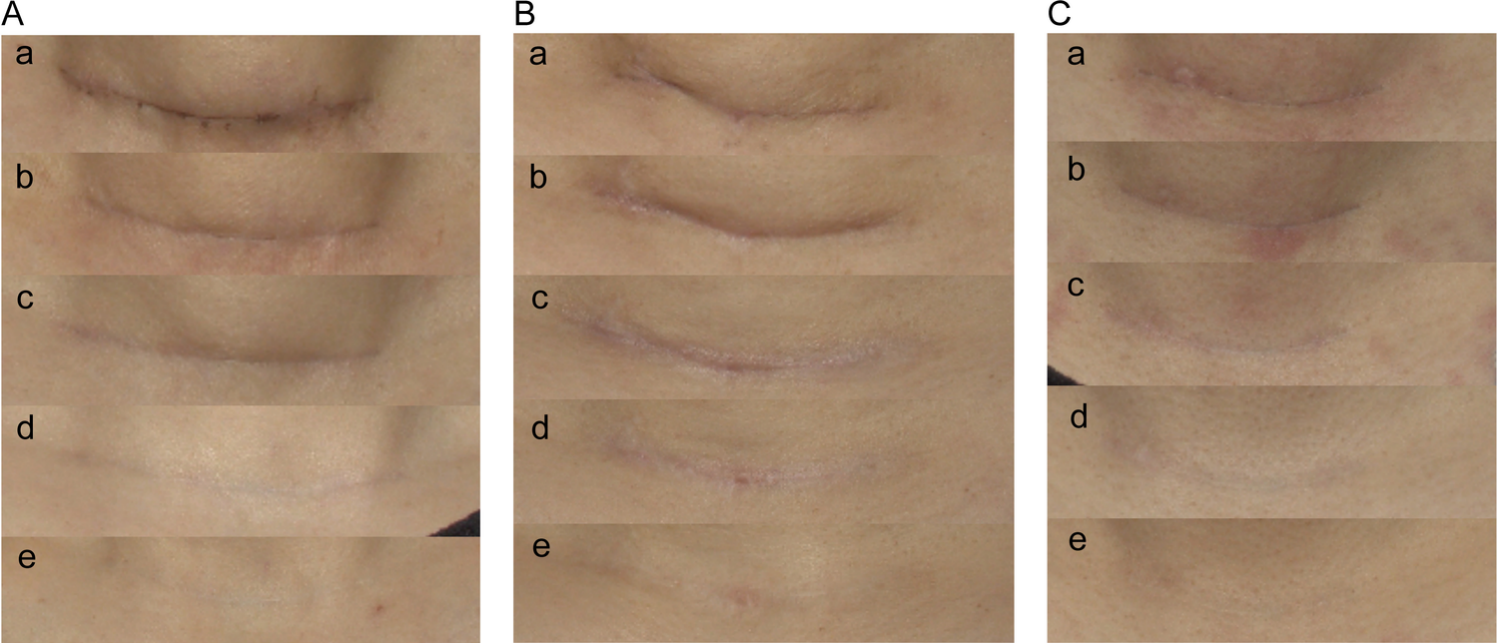
Representative scar healing progression showing best-case scenarios across treatment groups during 12-month follow-up period. **A** Group A (silicone dressing), **B** Group B (CO_2_AFL-IPL), **C** Group C (BTX-A). Time points: **a** baseline, **b** 1 month, **c** 3 months, **d** 6 months, **e** 12 months postoperatively. Photographic conditions were identical to those described for Fig. 3

At baseline, no significant differences in scar assessment parameters were observed among the three groups (*p* > 0.05). During follow-up, group differences gradually emerged: OSAS and mVSS showed significance at 1 month and became more pronounced at 3 months. By 6 months, most parameters demonstrated significant intergroup differences, and these differences remained significant at 12 months. Representative cases showing the dynamic progression of scar healing—from the worst- to best-case outcomes—are presented in Figs. 3, 4 and 5.

**Fig. 6:**
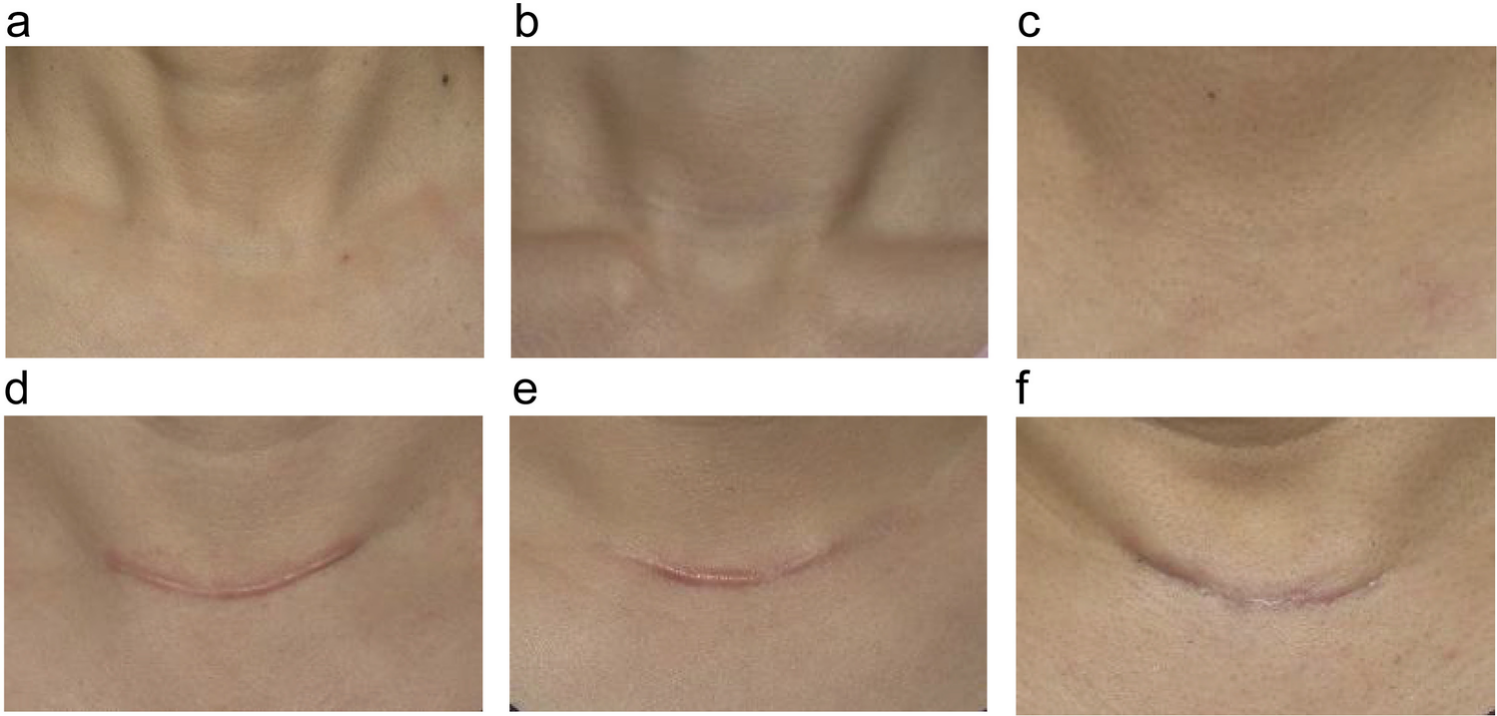
Case images of 12-month postoperative photographs of each group. In cases of best results, all groups achieved satisfactory scar outcomes. In general, however, both Groups B and C demonstrated superior scar quality compared to Group A, even in worst-evaluated cases. **a**, **b**, **c** Photographs of the best-evaluated patient in each group. (**a**, Group A; b, Group B; and c, Group C). **d**, **e**, **f** Photographs of the worst-evaluated patient in each group. (**d**, Group A; e, Group B; and f, Group C). Photographic conditions were identical to those described for Fig. 3

Primary endpoint analysis revealed significant differences in 6-month POSAS scores among the groups (*p* = 0.001). Both Group B (25.40 ± 8.11) and Group C (28.44± 15.40) achieved significantly better outcomes than Group A (37.52 ± 14.40), with no significant difference between Groups B and C. At 12 months, the treatment superiority of Groups B and C over the control group persisted (*p* < 0.001). All secondary endpoints followed similar trends, favoring both treatment modalities over the control at 12 months (*p* < 0.01). The mean imputation sensitivity analysis for missing data yielded results consistent with the primary analysis. Notably, even in the poorest outcomes, Groups B and C maintained better scar quality than Group A, as illustrated in representative photographs (Fig. 6). Sensitivity analyses yielded similar results: All outcomes that were statistically significant in the primary analysis remained significant, supporting the robustness of these findings.

### Scar Color Measurements

Scar color was assessed using a tristimulus colorimeter (DSM-4; Cortex Technology, Hadsund, Denmark) to evaluate the erythema index (EI), melanin index (MI), and gloss index, with additional CIE Lab* color space measurements (Table 3). From baseline to 12 months post-treatment, all groups demonstrated a decreasing trend in EI, with Group B showing the most pronounced reduction. MI gradually improved across all groups, whereas the gloss index increased consistently, reflecting enhanced scar surface texture. Although Group B showed more favorable changes in most parameters, intergroup differences were not statistically significant (*p* > 0.05).

**Table 3:** Scar colorimeter assessment results at different follow-up time points

Variables	Baseline			*P*	1-month			*P*	3-month			*P*
	Control group	CO_2_AFL-IPL group	BTX-A group		Control group	CO_2_AFL-IPL group	BTX-A group		Control group	CO_2_AFL-IPL group	BTX-A group	
E	22.44 ± 4.55	21.08 ± 5.38	20.43 ± 4.33	0.204	20.02 ± 5.58	19.28 ± 5.57	18.84 ± 4.02	0.618	18.33 ± 6.01	19.57 ± 4.27	17.78 ± 4.52	0.311
M	40.46 ± 3.15	39.49 ± 3.78	39.73 ± 4.11	0.521	38.98 ± 3.66	40.19 ± 4.77	39.63 ± 3.65	0.460	38.90 ± 3.36	39.17 ± 4.68	38.15 ± 2.85	0.495
Gloss	0.26 (0.00,1.71)	0.51 (0.00,1.50)	0.33 (0.00,2.38)	0.760	0.63 (0.04,2.05)	0.32 (0.06,1.38)	1.60 (0.42,3.71)	0.060	1.82 (0.22,3.13)	1.60 (0.17,4.57)	1.84 (0.47,3.77)	0.899
CIEL	53.62 ± 3.48	55.25 ± 3.63	55.59 ± 3.58	0.051	56.25 ± 4.24	55.94 ± 4.03	56.24 ± 3.42	0.930	57.25 ± 4.11	56.47 ± 4.13	57.85 ± 3.16	0.326
CIEa	9.61 ± 2.04	9.23 ± 2.72	8.51 ± 2.19	0.137	8.57 ± 2.73	8.20 ± 2.81	8.10 ± 1.97	0.715	7.70 ± 2.87	8.30 ± 2.07	7.44 ± 2.19	0.309
CIEb	15.80 ± 2.23	15.70 ± 1.74	16.69 ± 2.14	0.091	14.48 ± 1.70	14.69 ± 1.82	15.07 ± 1.52	0.338	13.46 ± 1.78	14.25 ± 1.80	13.84 ± 1.57	0.163

Variables	Baseline			*P*	6-month			*P*	12-month			*P*
	Control group	CO_2_AFL-IPL group	BTX-A group		Control group	CO_2_AFL-IPL group	BTX-A group		Control group	CO_2_AFL-IPL group	BTX-A group	
E	22.44 ± 4.55	21.08 ± 5.38	20.43 ± 4.33	0.204	16.94 ± 4.68	17.78 ± 4.77	17.59 ± 4.01	0.735	16.14 ± 3.69	14.88 ± 3.78	15.89 ± 4.41	0.469
M	40.46 ± 3.15	39.49 ± 3.78	39.73 ± 4.11	0.521	37.89 ± 3.19	38.57 ± 3.13	36.96 ± 2.85	0.093	37.61 ± 3.29	36.58 ± 2.33	37.20 ± 3.29	0.452
Gloss	0.26 (0.00,1.71)	0.51 (0.00,1.50)	0.33 (0.00,2.38)	0.760	1.39 (0.49,4.17)	1.88 (0.21,3.56)	1.69 (0.24,4.24)	0.858	2.74 (0.24,4.05)	2.29 (0.73,3.94)	1.40 (0.47,3.33)	0.575
CIEL	53.62 ± 3.48	55.25 ± 3.63	55.59 ± 3.58	0.051	58.53 ± 3.66	57.67 ± 3.44	58.68 ± 3.02	0.407	59.04 ± 3.37	60.10 ± 2.80	59.43 ± 3.44	0.481
CIEa	9.61 ± 2.04	9.23 ± 2.72	8.51 ± 2.19	0.137	6.97 ± 2.19	7.31 ± 2.41	7.29 ± 1.99	0.784	6.51 ± 1.76	5.87 ± 1.81	6.28 ± 2.21	0.474
CIEb	15.80 ± 2.23	15.70 ± 1.74	16.69 ± 2.14	0.091	13.23 ± 1.89	13.50 ± 1.93	13.65 ± 1.68	0.642	13.59 ± 2.24	13.90 ± 1.89	13.14 ± 1.99	0.387

### 3D Image Analysis

Scar volume was measured using the Vectra H2 3D Imaging System at all follow-up visits (Table 4). Volume reduction was observed across all treatment groups throughout the study period, reflecting the overall therapeutic effect on scar remodeling. In the early follow-up periods, all groups demonstrated similar volume reductions, with no significant intergroup differences (*p*> 0.05). At subsequent follow-ups, both treatment groups exhibited numerically greater reductions than Group A, although statistical significance was not reached. Notably, the trend toward greater volume reduction in the treatment groups became more pronounced at subsequent follow-up periods, suggesting the potential long-term benefits of the interventions.

**Table 4:** 3D Scar volume measurements at different follow-up TIME points

3D-scar volume	Control	CO_2_AFL-IPL group	BTX-A group	*P* value
1-month	− 2.57 (− 5.43,− 0.08)	− 3.56 (− 6.14,1.07)	− 2.62 (− 5.32,− 0.14)	0.963
3-month	− 5.58 (− 6.47,− 0.64)	− 4.18 (− 7.01,− 1.09)	− 5.19 (− 6.68,− 2.75)	0.669
6-month	− 4.19 (− 7.36,0.32)	− 5.78 (− 8.74,− 3.37)	− 5.14 (− 9.28,− 2.39)	0.260
12-month	− 4.39 (− 9.52,1.18)	− 6.10 (− 9.74,− 2.52)	− 5.95 (− 11.02,− 4.44)	0.285

### Safety Assessment and NRS Score

All 105 patients completed the treatments without experiencing serious adverse reactions. Group B showed a significantly higher incidence of adverse event than Groups A and C (*p *= 0.018), with post-inflammatory hyperpigmentation being the most frequent event, occurring exclusively in this group (4/35, 11.4%) (Table 5). Minor adverse events did not differ significantly among the groups. Pain assessment using NRS revealed a significant difference between Groups B and C (*p *= 0.002). Most patients in Group C reported mild pain (91.4% scored 1–3), whereas Group B exhibited a broader pain distribution (Table 6).

**Table 5:** Adverse events and safety assessment by treatment groups

Adverse reaction type	Control group	CO_2_AFL-IPL group	BTX-A group	*P* value
Bruising	0	1	1	1.000
Itching	1	1	0	1.000
Skin infection	0	1	0	1.000
Pigmentation	0	4	0	0.033
Total/Incidence rate	1	7	1	0.018

**Table 6:** Pain assessment (NRS score) during treatment procedures

NRS Score	CO_2_AFL-IPL group	BTX-A group
0	0	1
1-3	23	32
4-6	11	2
7-10	1	0
*P*-value	0.002	

## Discussion

This 12-month follow-up study provides the first direct comparison of BTX-A injection and a combined CO_2_AFL-IPL regimen for preventing post-thyroidectomy scars. At 6 and 12 months postoperatively, both interventions significantly outperformed silicone dressing alone on mVSS, OSAS, and POSAS, with no significant differences between the two treatments. The clinical meaning of these differences should be interpreted cautiously because no universally accepted minimal clinically important difference (MCID) exists for these scales. The extended follow-up was valuable in capturing scar maturation dynamics; notably, the superiority of both treatments persisted at 12 months, indicating sustained benefit throughout remodeling. Case examples spanning worst- to best-case outcomes likewise showed better scar quality with either active treatment versus control, supporting evidence-based selection of preventive strategies and informing standardization of post-thyroidectomy protocols.

We initiated treatment at 2 weeks postoperatively based on scar pathophysiology and consensus favoring early intervention, although the ideal window remains debated [2, 16]. By two weeks, re-epithelialization is complete, inflammation begins to subside, and remodeling is not yet established—conditions that may enhance responsiveness to therapy. Recent systematic reviews and meta-analyses support early CO_2_ fractional laser initiation, with treatment within the first postoperative month reducing scarring, whereas initiation after 3 months shows limited benefit [17]. This evidence underpins our decision to start CO_2_AFL-IPL treatment at 2 weeks. For BTX-A, efficacy has been reported with intraoperative and postoperative timing, with immediate intraoperative injection often superior [18, 19]. Dose can also be individualized: 5 U/0.1 mL yields optimal outcomes in high-risk regions, and 1 U/0.1 mL may suffice for low-risk areas [20], though timing protocols remain inconsistent across studies [21]. Our protocol—30 U BTX-A at 2 weeks—balanced individualized dosing by incision length and risk while respecting patient autonomy by avoiding immediate intraoperative injection for ethical reasons.

Mechanistically, silicone preparations may stabilize mast cells by maintaining hydration, modulating inflammatory cytokines, and preserving epidermal barrier homeostasis. BTX-A improves scar outcomes through combined molecular and biomechanical mechanisms. In addition to reducing acetylcholine-mediated muscle contraction and wound-edge tension, BTX-A has been shown to modulate fibroblast activity and profibrotic signaling, including downregulation of TGF-β1 and inhibition of the TGF-β/Smad pathway, thereby promoting more organized extracellular matrix remodeling and improved scar texture and contour [22–24]. Moreover, consistent with the chemoimmobilization concept, BTX-A may further improve scar predictability by attenuating local muscle activity and thereby reducing dynamic tension across the scar line, which is implicated in hypertrophic and keloid scarring [25–28]. This mechanism may be particularly relevant for post-thyroidectomy scars in the mobile anterior neck, where swallowing and phonation can increase tensile loading across a linear incision. Compared with the single pharmaceutical approach of BTX-A (reducing muscle tension and downstream profibrotic signaling), the laser combination targets multiple pathways [29]. CO_2_ fractional laser (10,600 nm) creates microthermal treatment zones that stimulate collagen neogenesis and remodeling with rapid healing from surrounding intact tissue, improving texture and appearance; earlier initiation appears more effective than delayed treatment[30]. Narrow-spectrum IPL enhances vascular targeting while limiting nonspecific thermal damage, and initiation at 2 weeks may accelerate vascular remodeling [31]. However, laser-based therapy carries risks—post-treatment crusting, persistent erythema, edema, and, with excessive energy/density or simultaneous dual-laser use, blistering, infection, or paradoxical hypertrophy [32]. Accordingly, we employed relatively conservative parameters, particularly for CO_2_ fractional laser, to balance efficacy and safety. Future work should refine parameter individualization (energy density, coverage, and intervals) to maximize effect while minimizing adverse events.

An observed clinical phenomenon in this study was erythematous, edematous, and pruritic reactions at drain sites in some patients; one case of drain-related infection was followed by scar hypertrophy. This likely reflects mechanical irritation, prolonged healing, and microbial colonization driving macrophage activation, chronic inflammation, and TGF-β/Smad signaling, thereby promoting pathological scarring [33, 34]. This observation argues for more rigorous drain management—judicious duration, meticulous local care, and early identification of infection.

Our objective measures warrant nuanced interpretation. Although POSAS detected between-group differences, colorimetry (erythema and melanin indices) and 3D stereophotogrammetry showed favorable but nonsignificant trends. Differences in devices, anatomical sampling sites, and measurement protocols may underlie these discrepancies and merit methodological harmonization [35, 36]. The POSAS 3.0 patient scale, tailored for linear scars and incorporating “widening of scar margins,” may enhance sensitivity for thyroidectomy scars and supports scar-type-specific assessment [37]. Furthermore, ultrasound elastography demonstrated threshold effects linking tissue stiffness to scar activity scores, reinforcing the value of integrating subjective scales with objective tools [38]. Establishing standardized, thyroidectomy-specific objective protocols could improve reliability across centers.

From a practical standpoint, BTX-A offers advantages in efficiency, patient experience, and implementation. Injections typically require ~5 min versus 20–30 min for laser therapy, and, in our cohort, BTX-A yielded lower pain scores and excellent acceptance without serious adverse events. Laser therapy, while effective, carries a risk of post-inflammatory hyperpigmentation in Fitzpatrick III–IV populations [39]. Resource needs also differ: BTX-A is less equipment-dependent and has fewer technical barriers, facilitating uptake in varied clinical settings. The single-session nature contrasts with three laser sessions, reducing patient and system burden. Given comparable efficacy, these factors suggest that BTX-A may represent a practical first-line option in many scenarios, while CO_2_AFL-IPL remains valuable where laser infrastructure and expertise are available or when clinician/patient preference favors device-based therapy.

This study has limitations. The single-center design, while ensuring high procedural consistency through a uniform surgical team and thereby reducing variability in scar formation, may nonetheless limit generalizability to broader clinical settings; multicenter validation is therefore needed. Because CO_2_AFL-IPL was evaluated as a combined regimen, the present findings may not be directly generalizable to CO_2_AFL-only or IPL-only protocols or to substantially different parameter settings. In addition, without formal intraclass correlation analysis, inter-rater variability could affect subjective scores, although assessor training, blinding, and inclusion of 3D imaging and colorimetry help mitigate bias. Twelve months may still be insufficient to capture late remodeling, and current objective tools may miss subtle differences. Future studies should extend follow-up, recruit across centers, and incorporate more refined technologies such as elastography, alongside cost-effectiveness analyses.

In summary, individualized protocols informed by neck anatomy and scar risk—covering BTX-A dose strategies and laser parameter selection—can advance precise prevention of post-thyroidectomy scarring. BTX-A dosing can be titrated from conservative to intensive based on risk and anatomic features. Additional research on optimized combination strategies and long-term economic outcomes will further guide clinical decision-making and resource allocation.

## Conclusion

Both BTX-A injection and CO_2_AFL-IPL treatment demonstrated comparable efficacy in preventing post-thyroidectomy scar formation, each yielding significantly superior outcomes than silicone gel dressing alone. Considering treatment convenience, procedural efficiency, patient comfort, safety, and cost-effectiveness, BTX-A injections exhibit superior clinical practicality. Therefore, BTX-A injection appears more practical in routine clinical use, whereas CO_2_AFL-IPL offers a valuable alternative for patients or institutions equipped for laser-based therapy.

## Data Availability

The data supporting the findings of this study are available from the corresponding author on reasonable request, if participant confidentiality is maintained.

## References

[1] Lyu Z, Zhang Y, Sheng C, Huang Y, Zhang Q, Chen K. Global burden of thyroid cancer in 2022: incidence and mortality estimates from GLOBOCAN. Chin Med J. 2024;137:2567–76. 39261986 10.1097/CM9.0000000000003284 PMC11557048

[2] Hong N, Sheng B, Yu P. Early postoperative interventions in the prevention and management of thyroidectomy scars. Front Physiol. 2024;15:1341287. 38523809 10.3389/fphys.2024.1341287 PMC10958159

[3] Yuan B, Upton Z, Leavesley D, Fan C, Wang XQ. Vascular and collagen target: A rational approach to hypertrophic scar management. Adv Wound Care. 2023;12:38–55. 10.1089/wound.2020.1348 PMC9595647 34328823

[4] Ogawa R. The most current algorithms for the treatment and prevention of hypertrophic scars and keloids: A 2020 update of the algorithms published 10 years ago. Plast Reconstr Surg. 2022;149:79e–94e. 34813576 10.1097/PRS.0000000000008667 PMC8687618

[5] Zhong S, Xiang Y, Xie H, Xiao J. Risk factors for scar formation after thyroidectomy and advances in its prevention and treatment. Aesthet Plast Surg. 2025. 10.1007/s00266-025-04883-z. 10.1007/s00266-025-04883-z 40295371

[6] Merdad M, Alqutub A, Magboul M, Awadh M, Faidah H, Aljohani AG, et al. Comparative aesthetic outcomes of wound closure methods following thyroidectomy: A systematic review and meta-analysis. Aesthet Plast Surg. 2025. 10.1007/s00266-025-05083-5. 10.1007/s00266-025-05083-5 40775191

[7] Bae DS, Koo DH, Kim JE, Cho J-m, Park J-O. Effect of Botulinum Toxin A on scar healing after thyroidectomy: A prospective double-blind randomized controlled trial. J Clin Med. 2020;9:868. 32245256 10.3390/jcm9030868 PMC7141531

[8] Wu WT. Skin resurfacing with Microbotox and the treatment of keloids. In: Botulinum toxins in clinical aesthetic practice. CRC Press; 2011. p. 204–19.

[9] Wu WTL. The microbotox technique. In: Tonnard P, Verpaele A, Bensimon R, editors. Centrofacial rejuvenation. New York, NY: Thieme Connect; 2018. p. 289–310.

[10] Radmanesh M, Mehramiri S, Radmanesh R. Fractional CO(2) laser is as effective as pulsed dye laser for the treatment of hypertrophic scars. J Dermatolog Treat. 2021;32:576–9. 31697183 10.1080/09546634.2019.1687821

[11] Su Q, Wang F, Chai Y, Yan Q, Wang F, Dong Z, et al. A prospective study on the treatment of immediate post-operative scar with narrowband intense pulsed light under polarized dermoscopy. J Cosmet Laser Ther. 2021;23:137–41. 35038956 10.1080/14764172.2021.2016844

[12] Chung JH, Kim DS, Cheon JH, Yoon JM, Baek SK, Jung KY, et al. Current protocol for aesthetic scar management in thyroid surgery. Laryngoscope. 2021;131:E2188-e2195. 33567135 10.1002/lary.29441

[13] Lv K, Xia Z. Chinese expert consensus on clinical prevention and treatment of scar. Burns Trauma. 2018;6:27. 30263894 10.1186/s41038-018-0129-9 PMC6154406

[14] Davey MG, Browne F, Davey MS, Walsh SR, Kerin MJ, Lowery AJ. Optimal primary wound closure methods after thyroid and parathyroid surgery: network meta-analysis of randomized clinical trials. BJS open. 2023;7(1):zrac170. 36821724 10.1093/bjsopen/zrac170 PMC9949711

[15] Lee K, Ward N, Oremule B, Mani N. Optimal wound closure techniques for thyroid and parathyroid surgery: A systematic review of cosmetic outcomes. Clin Otolaryngol. 2019;44:905–13. 31145548 10.1111/coa.13382

[16] Cuccolo NG, Tran DL, Boyd CJ, Shah AR, Geronemus RG, Chiu ES. Strategies for prevention and management of postoperative wounds and scars following microsurgical breast reconstruction: An evidence-based review. Adv Skin Wound Care. 2025;38:125–31. 40111065 10.1097/ASW.0000000000000282

[17] Ji Q, Luo L, Ni J, Pu X, Qiu H, Wu D. Fractional CO(2) laser to treat surgical scars: a system review and meta-analysis on optimal timing. J Cosmet Dermatol. 2025;24:e16708. 39780524 10.1111/jocd.16708 PMC11711943

[18] An MK, Cho EB, Park EJ, Kim KH, Kim LS, Kim KJ. Appropriate timing of early postoperative botulinum toxin type A injection for thyroidectomy scar management: a split-scar study. Plast Reconstr Surg. 2019;144:659e–68e. 31568312 10.1097/PRS.0000000000006064

[19] Phillips TJ, Fung E, Rigby MH, Burke E, Hart RD, Trites JRB, et al. The use of botulinum toxin type A in the healing of thyroidectomy wounds: a randomized, prospective, placebo-controlled study. Plast Reconstr Surg. 2019;143:375e–81e. 30688903 10.1097/PRS.0000000000005264

[20] Shi H, Zhang P, Zhang J, Sun J, Lv T. Dose-dependent effects of botulinum toxin type A on prevention of postoperative scars in various regions in the body: a prospective, double-blind randomized controlled trial. Aesthet Plast Surg. 2025;49:862–74. 10.1007/s00266-024-04351-0 39218835

[21] Chen Z, Chen Z, Pang R, Wei Z, Zhang H, Liu W, et al. The effect of botulinum toxin injection dose on the appearance of surgical scar. Sci Rep. 2021;11:13670. 34211099 10.1038/s41598-021-93203-x PMC8249595

[22] Baranowska A, Baranowska K, Czyżewski F, Filipek K, Kawka J, Maciek M, et al. Botulinum toxin type A in scar treatment: review article. Med Sci Basel. 2024;28:1–11.

[23] Li YH, Yang J, Zheng Z, Hu DH, Wang ZD. Botulinum toxin type A attenuates hypertrophic scar formation via the inhibition of TGF-β1/Smad and ERK pathways. J Cosmet Dermatol. 2021;20:1374–80. 33185943 10.1111/jocd.13842

[24] Lu J, Chen Y, Xie H, Wang Y. Botulinum toxin type A for preventing facial trauma and hypertrophic scars: a meta-analysis and trial sequential analysis. J Cosmet Dermatol. 2025;24:e70501. 41152696 10.1111/jocd.70501 PMC12569197

[25] Gassner HG, Sherris DA. Chemoimmobilization: improving predictability in the treatment of facial scars. Plast Reconstr Surg. 2003;112:1464–6. 14504533 10.1097/01.PRS.0000081073.94689.DB

[26] Gassner HG, Brissett AE, Otley CC, Boahene DK, Boggust AJ, Weaver AL, et al. Botulinum toxin to improve facial wound healing: a prospective, blinded, placebo-controlled study. Mayo Clin Proc. 2006;81:1023–8. 16901024 10.4065/81.8.1023

[27] Jablonka EM, Sherris DA, Gassner HG. Botulinum toxin to minimize facial scarring. Facial Plast Surg FPS. 2012;28:525–35. 23027220 10.1055/s-0032-1325641

[28] Sherris DA, Gassner HG. Botulinum toxin to minimize facial scarring. Fac Plast Surg: FPS. 2002;18:35–9. 10.1055/s-2002-19825 11823931

[29] Leszczynski R, da Silva CA, Pinto A, Kuczynski U, da Silva EM. Laser therapy for treating hypertrophic and keloid scars. Cochrane Database Syst Rev. 2022;9:Cd011642. 36161591 10.1002/14651858.CD011642.pub2 PMC9511989

[30] You HJ, Choi YS, Hwang NH, Kim DW, Oh KH, Kwon SY. The outcome of early ablative fractional laser treatment for thyroidectomy scars. Lasers Surg Med. 2020;52:721–9. 31950524 10.1002/lsm.23217

[31] Zhang Y, Ye R, Dong J, Bai Y, He Y, Ni W, et al. Efficacy and safety of ablative CO_2_ fractional laser and narrowband intense pulsed light for the treatment of hypertrophic scars: a prospective, randomized controlled trial. J Dermatolog Treat. 2023;34:2202287. 37070799 10.1080/09546634.2023.2202287

[32] Shin JU, Gantsetseg D, Jung JY, Jung I, Shin S, Lee JH. Comparison of non-ablative and ablative fractional laser treatments in a postoperative scar study. Lasers Surg Med. 2014;46:741–9. 25367640 10.1002/lsm.22297

[33] Yin J, Zhang S, Yang C, Wang Y, Shi B, Zheng Q, et al. Mechanotransduction in skin wound healing and scar formation: potential therapeutic targets for controlling hypertrophic scarring. Front Immunol. 2022;13:1028410. 36325354 10.3389/fimmu.2022.1028410 PMC9618819

[34] Yang N, Zhang H, Zhang Y, Lin B, Huang R, Cui T, et al. Bacterial colonization contributes to pathological scar formation via the regulation of inflammatory response. J Transl Med. 2025;23:569. 40400009 10.1186/s12967-025-06585-1 PMC12096502

[35] Kim BR, Kwon SH, Kim JW, Jeong WJ, Cha W, Jung YH, et al. Early postoperative injections of polydeoxyribonucleotide prevent hypertrophic scarring after thyroidectomy: a randomized controlled trial. Adv Wound Care. 2023;12:361–70. 10.1089/wound.2022.0025 35713247

[36] Kim JC, Choi JW, Kim YC. A prospective study to evaluate the treatment effect of pulsed dye laser on thyroidectomy hypertrophic scars using 3D imaging analysis. Lasers Surg Med. 2022;54:1082–8. 35842822 10.1002/lsm.23584

[37] Carrière ME, Mokkink LB, Tyack Z, Westerman MJ, Pijpe A, Pleat J, et al. Development of the patient scale of the patient and observer scar assessment scale (POSAS) 3.0: a qualitative study. Qual Life Res. 2023;32:583–92. 36355319 10.1007/s11136-022-03244-6 PMC9911488

[38] Hang J, Chen J, Zhang W, Yuan T, Xu Y, Zhou B. Correlation between elastic modulus and clinical severity of pathological scars: a cross-sectional study. Sci Rep. 2021;11:23324. 34857833 10.1038/s41598-021-02730-0 PMC8639709

[39] Mar K, Khalid B, Maazi M, Ahmed R, Wang OJE, Khosravi-Hafshejani T. Treatment of post-inflammatory hyperpigmentation in skin of colour: A systematic review. J Cutan Med Surg. 2024;28:473–80. 39075672 10.1177/12034754241265716 PMC11514325

